# Reduced efficiency of pelagic–benthic coupling in the Arctic deep sea during lower ice cover

**DOI:** 10.1038/s41598-023-33854-0

**Published:** 2023-04-25

**Authors:** Irina Zhulay, Katrin Iken, Paul E. Renaud, Ksenia Kosobokova, Bodil A. Bluhm

**Affiliations:** 1 Department of Arctic and Marine Biology, Faculty of Biosciences, Fisheries and Economics, UiT The Arctic University of Norway, Tromsø, Norway; 2 College of Fisheries and Ocean Sciences, University of Alaska Fairbanks, Fairbanks, USA; 3 Akvaplan-niva, Fram Centre for Climate and the Environment, Tromsø, Norway; 4 Department of Arctic Biology, University Centre in Svalbard, Longyearbyen, Norway; 5 Shirshov Institute of Oceanology, Russian Academy of Sciences, Moscow, Russia

**Keywords:** Ecosystem ecology, Stable isotope analysis, Environmental sciences, Marine biology

## Abstract

Pelagic–benthic coupling describes the connection between surface-water production and seafloor habitats via energy, nutrient and mass exchange. Massive ice loss and warming in the poorly studied Arctic Chukchi Borderland are hypothesized to affect this coupling. The strength of pelagic–benthic coupling was compared between 2 years varying in climate settings, 2005 and 2016, based on δ^13^C and δ^15^N stable isotopes of food-web end-members and pelagic and deep-sea benthic consumers. Considerably higher isotopic niche overlap and generally shorter isotopic distance were found between pelagic and benthic food web components in 2005 than in 2016, suggesting weaker coupling in the latter, low-ice year. δ^15^N values indicated more refractory food consumed by benthos in 2016 and fresher food reaching the seafloor in 2005. Higher δ^13^C values of zooplankton indirectly suggested a higher contribution of ice algae in 2005 than 2016. The difference in pelagic–benthic coupling between these years is consistent with higher energy retention within the pelagic system, perhaps due to strong stratification in the Amerasian Basin in the recent decade. Weaker coupling to the benthos can be expected to continue with ice loss in the study area, perhaps reducing benthic biomass and remineralization capacity; monitoring of the area is needed to confirm this prediction.

## Introduction

The deep sea is considered the world's largest sink for biogenic carbon^1^. Important insights into the global carbon cycle can, therefore, be gained from understanding the processes connecting ocean surface and seafloor through dynamics of organic matter, nutrients and energy cycling, i.e. pelagic–benthic coupling, in deep-sea ecosystems^2,3^. Pelagic–benthic coupling is considered to be tight when organic matter from surface production sinks to the seafloor with little reworking in the water column. Conversely, in weakly coupled systems, most of the energy is retained in the pelagic realm with low inputs to the benthos. Organic fluxes to the seafloor and strength of pelagic–benthic coupling vary in different ocean regions and largely depend on biological and physical processes in the water column^4–7^. One of the least studied regions in terms of trophic structure and carbon flux is the Arctic Ocean Basin with its complex morphological features along the perimeter^8^.

The Arctic Basin region is characterized by strong seasonality, with seasonal, and regionally multiyear, ice cover and the polar night lasting for up to 6 months, jointly constraining light availability needed for primary production^9,10^. In addition, nutrient concentrations in the surface water are often low, mostly due to strong stratification^11^. Therefore, the amount of pelagic primary production in the Arctic Basin is among the lowest recorded in the world, with average estimates of 1 to 25 g C m^−2^ y^−1^ ^12,13^. In addition to phytoplankton, sea-ice algae can contribute significantly to total primary production in this region. For example, ice algae have been previously reported to contribute more than 50% to the total primary production in the Arctic Basin^14^, a fraction that is much higher than their contribution of 4–30% on seasonally ice-covered Arctic shelves^15,16^. Other nutrient inputs, such as from advection of terrestrial matter and shelf production^17,18^ or from large food falls^19,20^, may be of local importance but are overall minor contributions for the Arctic Basin as a whole^11^.

The combined primary production sources serve directly as food for ice-associated and pelagic fauna. Their grazing intensity, in turn, has a strong impact on the amount and composition of organic matter reaching the seafloor^21,22^. Specifically, high pelagic grazing efficiency leads to a decrease in sedimentation of fresh phytoplankton, while little grazing facilitates higher sedimentation of intact phytoplankton cells and aggregates to the deeper water layers. The particulate organic matter (POM) can settle out in the form of intact cells, phytodetritus, fecal pellets, zooplankton carcasses, and marine snow^2,7,8^. During the descent, the POM undergoes additional biodegradation by bacteria and heterotrophs^1,23^. The amount and quality of material reaching the seafloor also depend on the water depth, as stronger vertical flux attenuation is expected in deeper areas of the Arctic Ocean^24^. Indeed, only a very small portion of carbon produced at the surface is estimated to reach the bottom of the Arctic deep sea (1–10%)^1,25–27^. Thus, typically very little and largely reworked organic particles reach the benthic fauna in the central Arctic, although export of fresh ice algal production has occasionally been observed^28^. Therefore, benthic trophic pathways in deeper areas of the Arctic Ocean have generally been described as longer than in shallower regions, with up to five trophic levels recorded for benthic species in the very few published studies from Arctic and sub-Arctic deep-sea environments^8,29^.

The presence or absence of sea ice may alter the strength of pelagic–benthic coupling in the Arctic marine ecosystems. Based on work on Arctic shelf systems, pelagic–benthic coupling is traditionally considered tighter in areas where sea ice is present^30^, although extremely high particle flux to the seafloor has recently also been observed during low sea ice cover on the Chukchi Sea shelf^31^. Ice algal production is mostly represented by large-sized diatoms that contribute significantly to a relatively fast transport of undisturbed organic matter to the seafloor^32,33^. In areas where open-water conditions dominate, pelagic phytoplankton is often characterized by a higher proportion of dinoflagellates than present in the sea ice community that might be retained more efficiently in the upper water column^5,33^. Similar connections between sea ice presence and stronger pelagic–benthic coupling have been observed^28^ or modeled for the Arctic deep sea^34^. Knowledge of food webs and pelagic–benthic coupling in the Arctic deep sea is, however, very scarce (but see^8,27,35,36^) due to logistical challenges related to sampling (e.g., remoteness of the area, great depth, ice cover, weather conditions, and the very low density of benthic fauna), leading to few observations mostly scattered over different Arctic deep-sea areas with the majority of studies being a snapshot in time.

The Arctic sea ice cover, however, is undergoing significant thinning and decrease in extent^37–39^. This decline is due to the Arctic currently experiencing strong warming of about four times the global average air temperature^40^. Thinning of sea ice allows higher light penetration^41,42^ and increases in primary production in several areas, primarily on shelves, of the Arctic Ocean^43,44^. However, small-sized primary producers (e.g., flagellate species) are expected to dominate in warmer, fresher, and nutrient-poor water^45,46^, like the Beaufort Gyre^47^. Smaller phytoplankton cells are more resistant to sinking^46,48^. In addition, pelagic grazing pressure can increase in response to increased primary production^49^, as well as due to increased advection of zooplankton with Pacific and Atlantic water into the Arctic Ocean^50^, leading to higher retention of organic matter in the water column. Thereby, physical and biological alterations related to climate change can lead to a weakening of pelagic–benthic coupling and carbon sequestration in deep-sea sediments, and, therefore, decrease in benthic food supply. However, it has not yet been evaluated whether the strength of the coupling in the central Arctic has been modified as a consequence of climate change since this is difficult or impossible to determine because few or no baseline data are available from former years (but see^36^).

**Figure 1:**
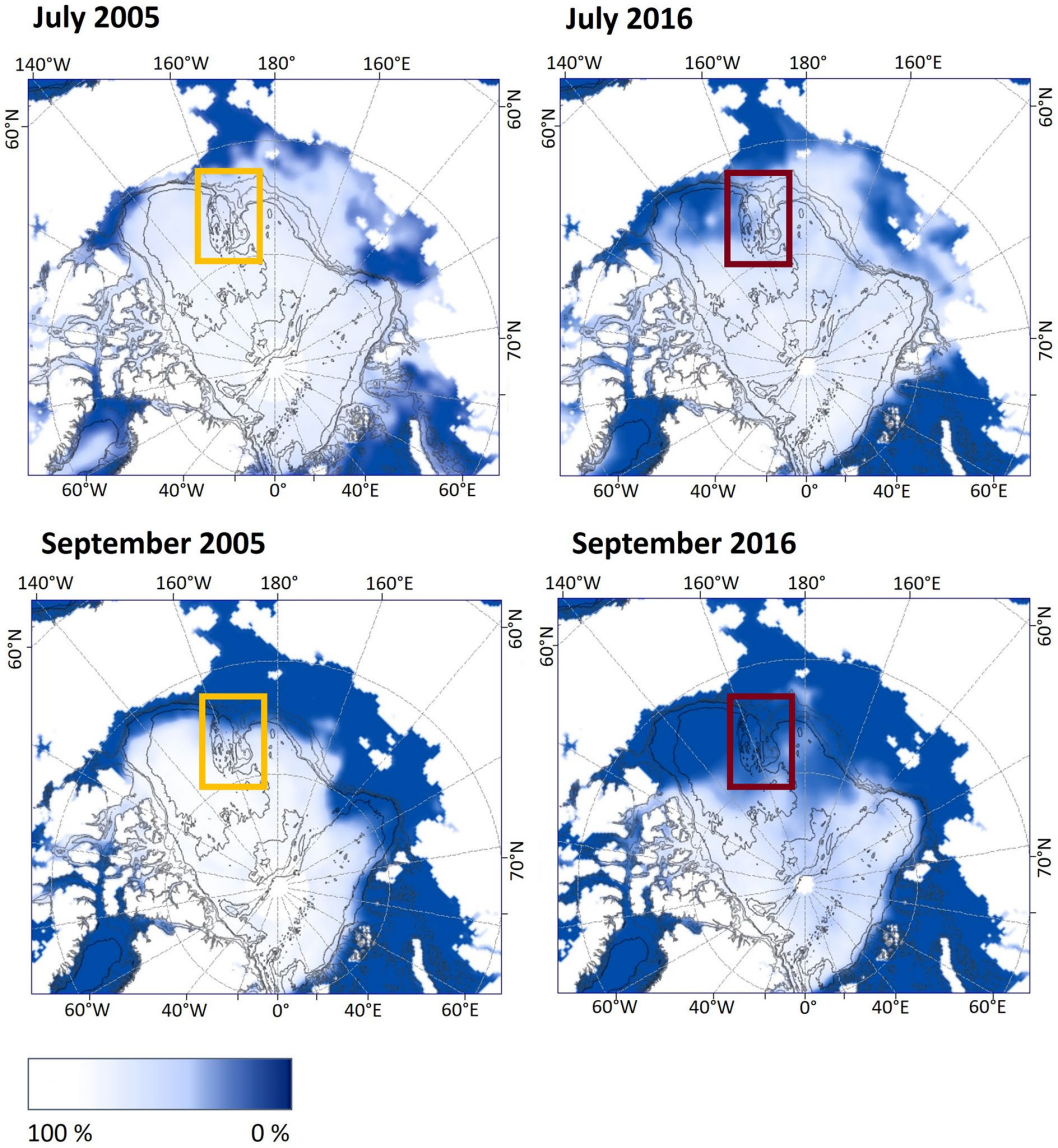
Comparison of sea ice concentration between the higher ice year, 2005 and the lower ice year, 2016, for July (upper panel, covers most of the sampling period for both years) and September (bottom panel, minimum ice month). The study area is marked by yellow and red rectangles for 2005 and 2016, respectively. Lowest ice concentrations are indicated by dark blue and highest concentrations are in white. Average sea-ice concentration data were derived from the National Snow and Ice Data Centre (https://nsidc.org/data/NSIDC-0051/versions/1). The data were then imported into ESRI ArcGIS 10.5 software (http://www.esri.com/software/arcgis/arcgis-for-desktop) and projected spatially.

**Figure 2:**
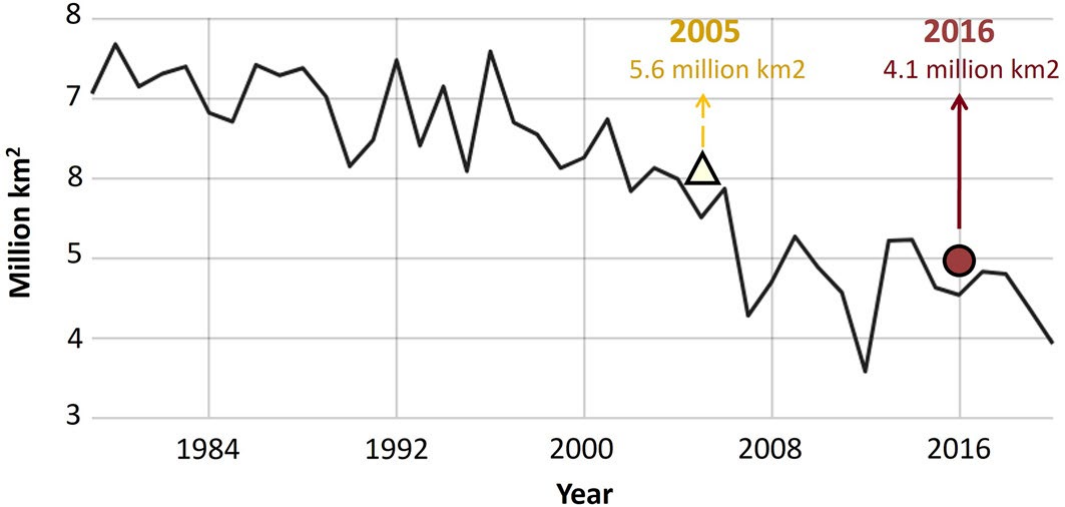
Arctic average September minimum sea ice extent (modified from  NASA Global Climate Change, NSIDC/NASA, climate.nasa.gov). Sampling years are indicated by a white triangle (2005) and red circle (2016), numbers above the triangle and circle indicate mean sea ice extent in 2005 (in yellow) and 2016 (in red).

In this study, we aim to assess potential changes in pelagic–benthic coupling in the Arctic Chukchi Borderland within the Canada Basin, where only few benthic studies on the topic have been conducted before^8,51–53^. While time series have been established on benthic biomass and food supplies, and coupling have been modeled on the adjacent Chukchi Sea shelf^54,55^, temporal comparisons in adjacent deep waters are lacking. We here consider 2 years characterized by different sea-ice settings—2005 and 2016 (Fig. 1), where we had the rare opportunity to perform repeat sampling at geographically close locations in the Arctic deep sea. While the Arctic system was already under the influence of lowered sea ice cover from climate change in 2005, signs of warming were much more pronounced by 2016^38^ (Fig. 2). Average sea-ice extent for September was ~ 6.9 million km^2^ until 2005, while it never exceeded 5.2 million km^2^ in the following years, including in 2016 when the September sea-ice extent was 4.1 million km^2,38,56,57^ (Figs. 1 and 2). In addition, a continuous decline in sea-ice thickness and, hence, increased dominance of first-year ice over multiyear ice, was registered over the last decades and including the period of our study^39,58^. We tested the hypothesis that pelagic–benthic coupling was tighter in the early 2000s when more sea ice was present (represented here by 2005) compared to later, lower ice years (represented by 2016). Following earlier studies on pelagic–benthic coupling in Arctic regions^8,59,60^, we used stable nitrogen and carbon isotope analysis of POM endmembers and pelagic and benthic consumers to investigate pelagic-benthic coupling, specifically by comparing food source use and trophic niche space between the 2 years. This approach is based on the well-established concept that nitrogen stable isotope ratios indicate trophic position of organisms as tissues are progressively enriched in the heavier isotope with increasing trophic level in a reasonably predictable manner^61^. Thus, lower δ^15^N values of benthic taxa can be expected in a food web where pelagic-benthic coupling is tight. Carbon stable isotope ratios in consumers are indicative of carbon endmember utilization based on different isotopic ratios of different primary producers or habitats^61,62^. For example, sea-ice algae are often enriched in ^13^C compared to phytoplankton (on average by 4–5‰, though highly variable)^63,64^. Thus, higher consumer carbon isotope values can be found in areas where ice algae are a main food source. Both trophic markers (^15^N and ^13^C) combined describe trophic niches in isotope biplot space^65^. A high overlap of isotopic niches of pelagic and benthic members in a given food web can indicate tight coupling between these two realms. Therefore, we hypothesized a decrease in pelagic-benthic coupling strength would be reflected in a lower overlap of pelagic and benthic isotopic niches, higher δ^15^N values of benthic organisms, as well as lower δ^13^C values in benthic consumers from reduced ice algal uptake associated with lower ice extent.

## Results

**Figure 3:**
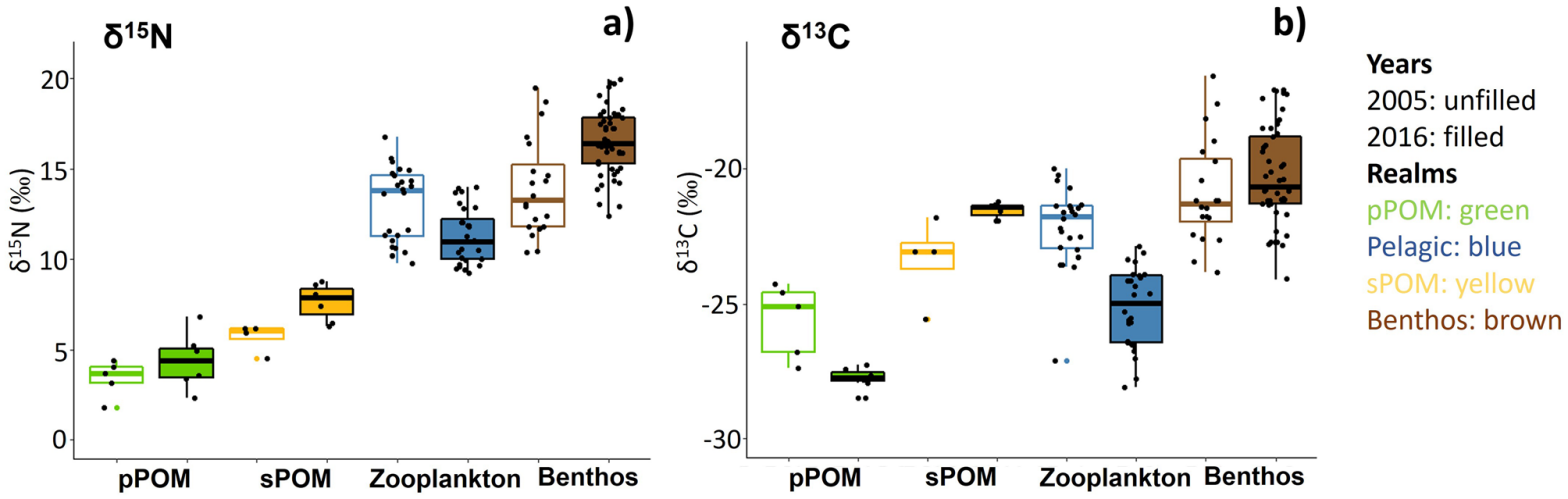
Mean values of (**a**) δ^15^N and (**b**) δ^13^C (‰) per food web component (pPOM in green, pelagic in blue, sPOM in yellow, and benthos in brown) in 2005 (open boxplots) and 2016 (filled boxplots), collected in the Chukchi Borderland. pPOM and sPOM is pelagic and sediment particulate organic matter, respectively. Only comparable taxa were included (i.e., either the same or closely related taxa).

Mean δ^15^N values of sPOM (sediment POM) and benthos (i.e., benthic invertebrates) collected in 2005 were significantly lower than those collected in 2016 (Fig. 3a, Table 1). The δ^15^N range of sPOM was 1.6‰ (4.5–6.2‰) in 2005 and over 2.5‰ (6.3–8.8‰) in 2016 (Tables S1, S3). The range of δ^15^N of benthic consumers was 9.1‰ (10.4–19.5 ‰) in 2005 and 8.1‰ (12.4–20.5‰) in 2016 (Tables S1, S3). Mean δ^15^N values of pPOM (pelagic POM), however, did not differ significantly between the 2 years, although mean δ^15^N of pPOM in 2005 was slightly lower than in 2016. The range of δ^15^N of pPOM was 3.7‰ (from 1.4 to 5.1‰) in 2005 and 5.7‰ (from 1.9 to 7.6‰) in 2016 (Tables S1, S3). In contrast, for zooplankton, the mean δ^15^N was significantly higher in 2005 than in 2016, with the range of δ^15^N of zooplankton values being 9.1‰ (between 8.1 and 17.2‰) in 2005 and 6.4‰ (between 8.3 and 14.7‰) in 2016 (Fig. 3a, Tables 1, S1, S3).

**Table 1:** Comparison of δ^15^N and δ^13^C (‰) values from different food web components in the Chukchi Borderland between 2005 and 2016: results of parametric two sample *t*-test (t), Welch’s two sample *t*-test (t) and Wilcoxon rank sum test (W) (for choice of test see methods).

	t	W	df	n	*p*-value
δ^15^N					
pPOM	1.25		10	12	0.24
sPOM	3.36		9	11	**0.008**
Zooplankton		510	–	52	**0.001**
Benthos	3.86		27	69	** < 0.001**
δ^13^C					
pPOM	3.95		10	12	**0.003**
sPOM		2	–	11	**0.029**
Zooplankton		630		52	** < 0.001**
Benthos	1.32		67	69	0.191

**Figure 4:**
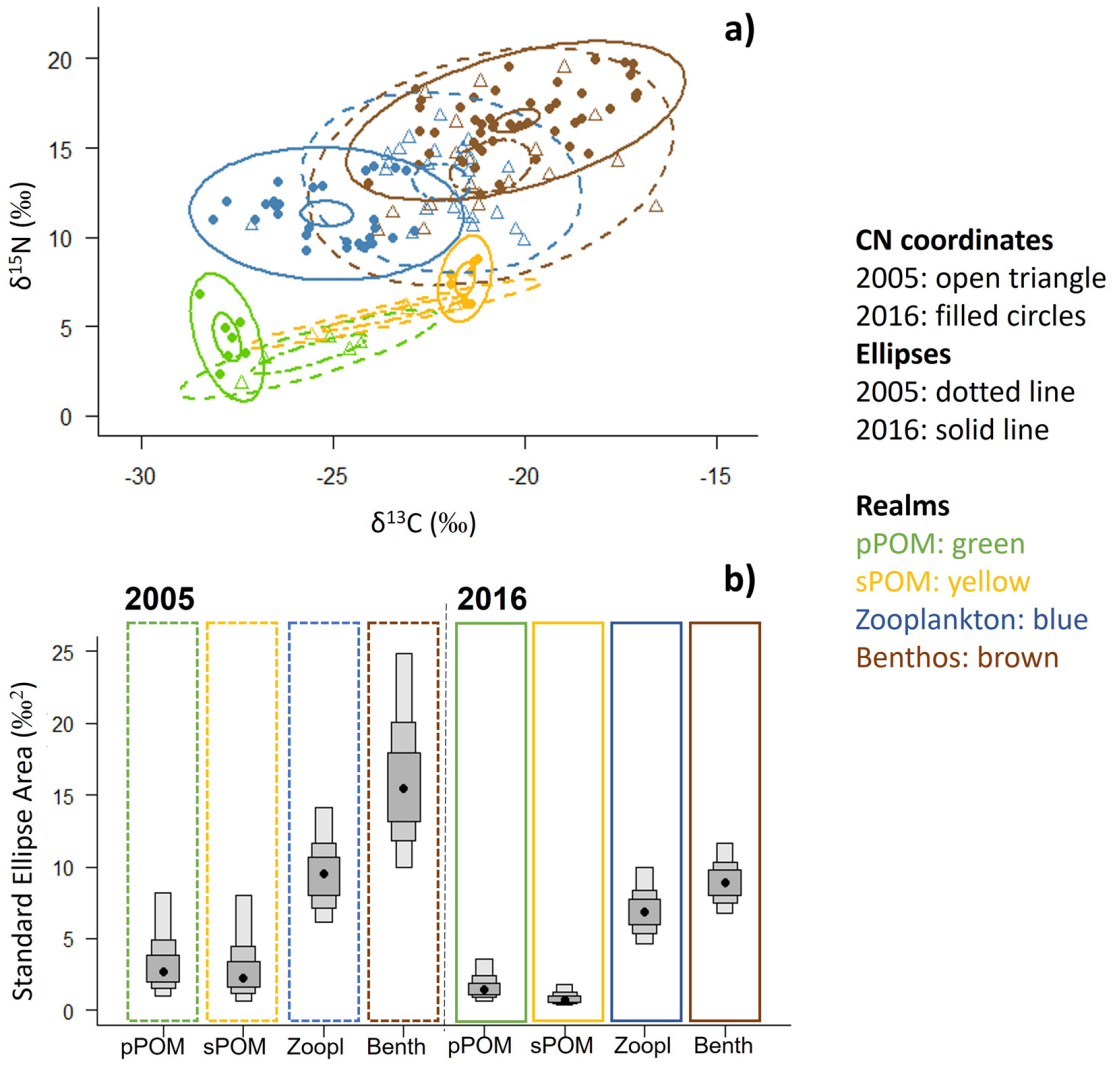
Isotopic niches of compared food web components in the study area. (**a**) Biplot of δ^13^C and δ^15^N isotope values (‰) for assemblages in the Chukchi Borderland in 2005 and 2016; outer ovals are sample size corrected standard ellipses (SEA_C_) containing 95% of the data. Inner small ovals indicate 95% confidence intervals around the bivariate means. Open triangles are means of samples collected in 2005 and filled circles in 2016. Food web endmembers and consumers are indicated by colors: green (pPOM), yellow (sPOM), blue (zooplankton), and brown (benthos). See Table 3 for pPOM and sPOM abbreviations. (**b**) Standard ellipse areas Bayesian estimations (SEA_B_) presented as credible intervals for each of the community components for the two sampling years; black dots are the mode of the SEA_B_ (‰^2^), the shaded boxes represent the 50% (dark grey), 75% (lighter grey) and 95% (lightest grey) credible intervals. Dotted outlines enclose assemblages collected in 2005 and solid lines represent those collected in 2016. Total amount of zooplankton taxa consisted five for both years, and total amount of benthic taxa consisted 13 in 2005 and 32 in 2016. Only comparable taxa were included (i.e., either the same or closely related taxa).

Numbers in bold indicate statistically significant results (*p* values < 0.05). See Table 3 for pPOM and sPOM abbreviations. See Fig. 4 for graphical representation of means and error.

**Table 2:** Isotopic distances between means of δ^15^N and δ^13^C (‰) of endmembers (pPOM, sPOM) and consumers (zooplankton, benthos) from the Chukchi Borderland in 2005 and 2016. See Table 3 for pPOM and sPOM abbreviations.

	Distance between				
	pPOM and sPOM	pPOM and zooplankton	pPOM and benthos	zooplankton and benthos	sPOM and benthos
δ^15^N					
2005	2.1	9.6	10.5	0.9	8.4
2016	3.3	6.9	12.1	5.2	8.9
δ^13^C					
2005	1.7	3.5	4.7	1.3	3.0
2016	6.2	2.6	7.5	4.9	1.3

Mean δ^13^C values of pPOM and zooplankton in 2005 were significantly higher than those in 2016 (Fig. 3b, Table 1). The δ^13^C range of pPOM was 3.7‰ (from − 24.0 to − 27.7‰) in 2005 and 2.9‰ (from − 28.9 to − 26.0‰) in 2016. The δ^13^C range of zooplankton comprised 8.1‰ (from − 27.8 to − 19.7‰) in 2005 and 6.9‰ (from − 28.6 to − 21.7‰) in 2016 (Tables S1, S3). Conversely, mean δ^13^C of sPOM was significantly lower in 2005 compared to 2016 (Fig. 3b, Table 1) and ranged 2.5‰ (from − 25.6 to − 23.1‰) in 2005 and 0.7‰ (from − 21.2 to 21.9‰) in 2016 (Tables S1, S3). There was no significant difference between the years for mean δ^13^C of benthic organisms (Fig. 3b, Table 1), and the δ^13^C values ranged over 8.2‰ (− 24.8 to − 16.6‰) and over 8.4‰ (− 24.6 to − 16.2‰) in 2005 and 2016, respectively (Tables S1, S3).

Isotopic niche size, measured as standard ellipse area (SEAc), of the benthic component differed between 2005 and 2016 with a wider niche in 2005 (Fig. 4a, b, Table S3). This difference was confirmed by high probability of difference (96%) between posterior Bayesian estimates of standard ellipse areas (SEA_B_) for the benthos components between years. Compared to the benthos, the isotopic niche size of zooplankton was more similar between years, with an 86% probability of difference; as this probability was below the threshold of 95%, zooplankton niche sizes were not considered statistically different (Fig. 4a, b, Table S3).

SEAc overlap between consumer groups and between endmembers was also different for the 2 years. Specifically, overlap between benthos and zooplankton was considerably higher in 2005 (57.9%) than in 2016 (5.5%). The SEAc overlap between sPOM and pPOM was generally low, but also higher in 2005 (4.8%) than in 2016, when the two SEAc did not overlap (Fig. 4a, Table S4).

δ^15^N isotopic distances between pairs of food web components were mostly smaller in 2005 compared to those in 2016 (Table 2). The exception was the isotopic distance between pPOM and zooplankton, which was higher in 2005 compared to 2016 (Table 2). For δ^13^C, the same trend of shorter isotopic distances between the food web components in 2005 versus 2016 was also evident between the following pairs: pPOM and sPOM, pPOM and benthos, zooplankton and benthos (Table 2). Conversely, δ^13^C isotopic distance was higher between pPOM and zooplankton, and sPOM and benthos in 2005 compared to 2016 (Table 2).

## Discussion

The degree to which water column and benthic processes are coupled influences benthic community composition, production, trophic structure, and elemental cycling rates^7,66,67^. This is particularly true in the energy-limited deep sea, where benthos is largely sustained by production originating in the surface-water layers^68^. Based on stable isotope data collected in the poorly studied Arctic Chukchi Borderland, we evaluated differences in pelagic-benthic coupling between 2 years characterized by different climate settings. In 2005, the ice cover was still comparatively high despite some evidence of regional warming^56^, while by 2016 the Arctic had experienced a series of very low sea ice years and undergone transformations due to climate change^38^. Results of our study suggested tighter pelagic-benthic coupling in 2005 than 2016, which generally supported our hypothesis. This difference was reflected in much higher overlap of zooplankton and benthic isotopic niches in 2005 than in 2016. Similarly, pelagic and benthic food-web endmembers slightly overlapped in 2005, while no overlap was observed in 2016. These findings are consistent with shorter δ^15^N and δ^13^C isotopic distances between pPOM and sPOM, pPOM and benthos, and zooplankton and benthos in 2005 compared to 2016.

Multiple mechanisms could underlie the patterns we found. Lower surface primary production in 2016 relative to 2005^69,70^ could explain pelagic-benthic coupling differences between the sampling years, as the level of primary production in part determines how much organic matter will eventually reach the seafloor. Although increased primary production has been observed in many areas of the Arctic Ocean over the last two decades^43,71,72^, low and in part declining values of primary production and/or Chl *a* concentration have, in fact, been documented^36,43,69,70^ or modeled^69,73^ in the Beaufort gyre zone and adjacent waters, including the Chukchi Borderland, during the last few years. The reduced primary production was primarily attributed to exceptionally high freshening of the Canada Basin^74,75^, resulting in strengthened stratification and inhibition of nutrient renewal in the euphotic zone^76,77^.

The source of primary production can also influence pelagic-benthic coupling. Based on the higher sea ice cover in 2005, we might assume that the abundance of ice algae was also higher in that year, though ice-algal biomass was not measured in the present study. In the adjacent northeastern Chukchi Sea, however, the ice algal signal at the seafloor, assessed by the isoprenoid trophic marker IP_25_, had declined between 2012 and 2017^78^. Consistent with this observation, our results showed significantly higher δ^13^C values of pPOM in 2005 than in 2016, which might indicate higher contribution of ice algae in 2005, as sea-ice algae are often enriched in ^13^C compared to phytoplankton^63,64^. However, δ^13^C values of ice POM from samples taken during the 2005 expedition did, for the most part, not differ from those of pPOM^79^ at the time of sampling. This absence of an isotopic difference may be due to high CO_2_ exchange between water, ice, and atmosphere when ice becomes more porous towards the summer^8,80,81^. Instead, the significantly higher δ^13^C values of zooplankton in 2005 compared to 2016 might be an indicator of consumption of ice POM produced *earlier* in the year during the ice-algal bloom when ice structure still restricted CO_2_ exchange. The isotopic turnover time between food and consumer of about three weeks for copepods in the Arctic^82^ makes it feasible that an earlier, enriched carbon isotope ice algal signal might be present in the zooplankton at our time of sampling in 2005. The potentially higher ice POM contribution in 2005 was, however, not reflected in the sediment and benthic tissue samples, unlike observed in other studies^64,80^. δ^13^C values of benthos did in fact not differ significantly between years, and δ^13^C of sPOM was significantly lower in 2005 than in 2016, though the sample size of sPOM was low for both years. Our data cannot resolve whether ice POM did not reach the seafloor (because it was consumed in transit), was too patchy to be captured by our sampling, or in fact was not isotopically enriched enough to be visible in benthic taxa and sPOM. In summary, some evidence points to the possibility of ice algae playing a role in the apparent difference in pelagic-benthic coupling between the study years, but unequivocal conclusions are difficult based on a single sampling period in each year.

Besides the amount and sources of primary production, freshness and, hence, quality of food has an effect on benthic trophic structure^23^. Mean δ^15^N values of sPOM and benthos were significantly lower in 2005 compared to 2016, which indicates that organic matter available to the benthos was generally less reworked in 2005 than in 2016. In addition, the isotopic niche of benthos was significantly wider in 2005 than in 2016, even though fewer benthic samples were available in 2005 (Tables S1, S2). The difference in isotopic niche of the benthos was essentially driven by a larger δ^15^N range in 2005 compared to mainly high δ^15^N values in 2016. This upper range of consumer values in 2016 is also included in the benthic niche in 2005, suggesting that the same carbon was available for food in 2005, along with a 'fresher' source characterized by benthic consumers with a lower δ^15^N ratio. Potential differences in food quality might be related to decreased relative contribution of large phytoplankton and ice algae (diatoms) and increased contribution of small cells (such as flagellates) in 2016 related to sea ice loss^38,83^, freshening of the area^48,70^, and strengthened stratification in recent years^11^. As a result, vertical organic matter export flux would have been dominated by faster sinking and, thus, fresher food sources^28,84,85^ for benthic consumers in 2005 than 2016. While we lack direct evidence for this hypothesis from our region, a study from deep Arctic Fram Strait indeed supplies indirect evidence in that the authors documented higher organic matter export efficiency in regions with than without seasonal sea ice^86^.

Further, the strength of pelagic-benthic coupling is affected by grazing efficiency of zooplankton, which largely depends on the density, species composition, and developmental stages of herbivorous zooplankton present at the time of primary production. At high zooplankton densities and grazing rates, downward carbon flux can be reduced^34^ which might be expected if zooplankton densities increased with stronger advection of Pacific species into the basin or perhaps by locally increased reproductive output^35,50^. The few available inter-annual zooplankton studies from the Canada Basin region^87,88^, however, do not suggest a trend for increasing zooplankton populations between 2007 and 2017, nor do Abe et al.’s^88^ model results imply zooplankton increases in the region in the study period.

## Summary and conclusion

Evaluation of climate change effects on pelagic-benthic coupling in the deep Arctic Ocean is difficult due to limited availability of long-term data sets^36^. In the present study, we compared pelagic-benthic coupling in 2005, at the end of a decade with only early signs of warming^56^, and 2016, when years of intense climate warming had been documented and impacts on system drivers were observed. Our results suggest stronger coupling of benthic and pelagic realms in 2005 compared to 2016 and may indicate that ice-algal contribution was potentially higher in zooplankton diets in 2005 compared to 2016. This inference is consistent with observations from the nearby NE Chukchi Sea shelf and comparisons of vertical carbon export in ice-covered versus open water areas in deep Fram Strait, yet seasonal sampling in our study area would have been needed to provide firm evidence. Benthic communities received fresher organic material in 2005 than in 2016, as evidenced by δ^15^N values of benthic consumers and sPOM. The inferred decoupling in 2016 is consistent with physical and biological changes that were observed in the region in recent years. Specifically, a shift from perennial to seasonal sea ice^38,39^ may have resulted in an overall shift in primary producer composition and vertical carbon export within this system. Strengthening of the halocline within this region^89^ has resulted in a decrease in primary production in the area^69^ and a shift to small-celled phytoplankton^70^. We propose that these changes likely lead to a longer residence time of organic matter in the water column, a higher level of organic matter biodegradation before it reaches the seafloor, and, thus, a decrease in overall organic matter flux to the seafloor. This situation would reduce carbon storage in deep-sea Arctic benthos. Since ecosystem responses to climate change varies depending on local environmental and biological settings, it is recommended that time-series observations, similar to those on the adjacent Chukchi Sea shelf^54^, be extended into the deep Arctic Ocean basin.

## Materials and methods

### Sea ice situation

To illustrate the difference in sea-ice cover between the sampling years, we plotted average sea-ice concentration data derived from satellite Nimbus‐7 SMMR and DMSP SSM/I‐SSMIS Passive Microwave at a grid cell size of 25 × 25 km^90^ for both study years. The concentration is defined as the fraction of the area of the grid cell covered by sea ice and is given in percentage from 0 (no ice) to 100 (fully covered by ice) percent ice (https://nsidc.org/cryosphere/seaice/data/terminology.html). Average sea ice concentration for July and September (minimum ice month) was downloaded from the National Snow and Ice Data Centre (https://nsidc.org/data/NSIDC-0051/versions/1). The data were then imported into ArcGIS 10.5^91^ software and projected spatially.

### Sample collection

**Figure 5:**
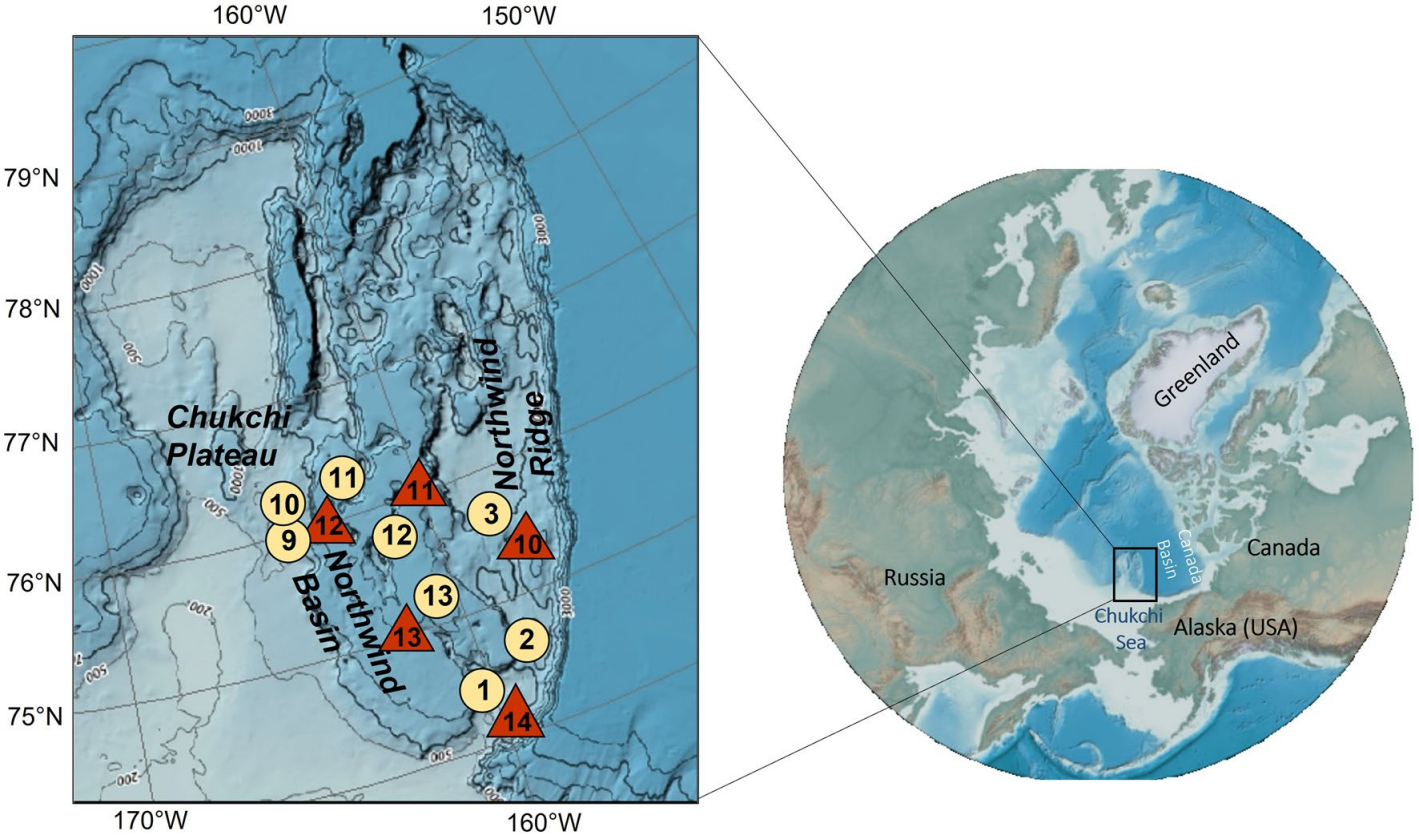
Study area and stations sampled in the Arctic Chukchi Borderland. Stations sampled in 2005 and 2016 are indicated by yellow circles and red triangles, respectively. Numbers in bold print are station numbers; small numbers along isobaths indicate water depth. The map was created using ESRI ArcGIS 10.5 software (http://www.esri.com/software/arcgis/arcgis-for-desktop).

To assess pelagic-benthic coupling, samples were collected during the “Hidden Ocean” expeditions onboard the US Coastguard icebreaker *HEALY* between 28 June and 25 July 2005 (expedition HLY05-02) and 2 July and 10 August 2016 (expedition HLY16-01) (Table 3). Sampling stations were located between 74–76°N and 158–163°W (Fig. 5, generated using ArcGIS^91^). In 2005, five stations were sampled at depths ranging from 621 to 2090 m (Table 3, Fig. 5). In 2016, eight stations were sampled at depth varying between 486 and 2107 m (Table 3, Fig. 5).

**Table 3:** Station locations, depth and sample types collected for stable isotope analysis in 2005 and 2016 in the Chukchi Borderland of the Arctic Ocean. pPOM and sPOM is pelagic and sediment particulate organic matter, respectively.

Year	Station	Depth (m)	Latitude (°N)	Longitude (°W)	Zooplankton	Benthos	pPOM	sPOM
2005	10	621	75.46	158.32	X		X	
	11	1,374	76.01	160.41	X	X	X	X
	12	937	76.26	163.29	X	X	X	X
	13	2,090	75.16	161.13	X	X	X	X
	14	749	74.18	159.54	X		X	
2016	1	853	74.37	159.53	X	X	X	X
	2	1,059	74.66	158.38	X	X	X	X
	3	746	75.68	158.53	X	X	X	X
	9	508	76.51	163.78		X	X	X
	10	873	76.41	163.56	X	X	X	X
	12	2,107	75.73	161.24	X	X	X	X
	13	2,091	75.23	160.38	X	X	X	X

Pelagic POM (pPOM) was collected at each station in both years from the chlorophyll maximum layer from Niskin bottles attached to a SBE9/11 + CTD rosette equipped with an in-situ fluorometer. The chlorophyll maximum layer varied from 30 to 60 m depth in 2005 and from 50 to 70 m in 2016. Two to three water samples per station were collected from different bottles of the same CTD cast, totaling 13 pPOM samples in 2005 and 21 pPOM samples in 2016 (Table 3, Table S1). The collected water samples were then filtered onto pre-combusted, 25 mm diameter GF/F filters. Large organisms visible by eye on the filters (e.g., meso-zooplankton) were removed.

Sediment POM (sPOM) was collected into a sterile plastic bag from the top ~ 1 cm sediment from 0.06 m^2^ box core samples at 3 stations in 2005 and from 0.25 m^2^ box core samples at each of the 7 stations in 2016 (Table 3). One replicate sediment sample per station was collected per year (Table S1).

Zooplankton consumers were collected at 5 stations with a multi-net (Midi, Hydrobios, 150 μm) in 2005 and at 6 stations with the same Multinet in 2016 (Table S1). Five zooplankton species common to the upper water column in the Arctic Basin and representing different taxonomic groups with different food preferences were chosen for the analysis: the copepods *Calanus glacialis* (grazer), *Calanus hyperboreus* (grazer), *Paraeuchaeta glacialis* (predator), the amphipod *Themisto abyssorum* (predator/omnivore), and the chaetognath *Eukrohnia hamata* (predator, but see^92^). Often, mass of individual zooplankton organisms was insufficient for isotopic analysis; thus, several individuals of the same species were pooled by station. A total of 71 zooplankton samples were collected in 2005 and 66 in 2016. Replication varied from 1 to 3 samples of each zooplankton species per station (Table S1).

Epifaunal benthic consumers, including some demersal fish, were sampled with a 7 mm mesh (4 mm cod end) beam trawl and a Remotely Operated Vehicle (ROV *Global Explorer*, Deep-Sea Systems Inc. in 2005, and Oceaneering International in 2016) in both years. The ROVs were equipped with a suction hose and a manipulator arm enabling targeted sample collection. Infaunal benthic consumers were collected with a 0.25 m^2^ box core in both years. All benthic samples were washed to remove sediments (2 mm mesh size for beam trawl, 0.3 mm for box core samples) and fauna were identified to the lowest taxonomic level possible. Vouchers of invertebrate taxa were collected when identification was uncertain and identified later by experts (see acknowledgments). Taxon names were verified with WoRMS (www.marinespecies.org, 30.12.2022). Benthic consumers were then subsampled for muscle tissue, where possible, to represent a tissue with slow turnover rate^93^. Where muscle tissue was not distinguishable or unavailable, tissue was sampled from body walls (e.g., anemones), tube feet (e.g., asteroids), and entire organisms were collected when body mass was small (e.g., some worms, small amphipods). A total of 29 and 85 benthic organism samples were collected in 2005 and 2016, respectively, with replication varying from 1 to 3 per species per station (Table S1). All samples collected for isotope analysis were frozen at − 20 °C immediately after collection until laboratory analyses.

### Laboratory analysis

pPOM filters were fumed with concentrated hydrochloric acid (HCl) vapor for 48 h and dried before analysis. sPOM samples were thawed and each sample was homogenized by mixing. Approximately 1 ml of the sediment was treated with 1 N HCl until bubbling stopped and then rinsed with distilled water until pH of the sediments was close to neutral, after which the samples were freeze-dried before analysis^18,60^. All organism tissue samples were dried at 60 °C for 24 h prior to the laboratory analysis. Lipids in zooplankton tissue samples were removed with repeated use of a 2:1 ratio of chloroform:methanol to avoid interpretation bias in lipid-rich zooplankton^94^. The samples were then re-dried at 60 °C for 24 h. Tissue samples that contained high carbonate concentrations were acidified with 1 N HCl for carbon isotope analyses to prevent the bias introduced by inorganic carbon in δ^13^C values. The acid was removed by rinsing with distilled water after bubbling had ceased; then, samples were dried again at 60 °C for 24 h.

All carbon and nitrogen stable isotope analyses were performed at the Alaska Stable Isotope Facility at the University of Alaska Fairbanks on a Thermo Finnigan Delta Isotope Ratio Mass-Spectrometer with Vienna PDB as standard for carbon and atmospheric N_2_ as standard for nitrogen. Instrument error was < 0.2 ‰ for δ ^13^C and < 0.4 ‰ for δ ^15^N in 2005, and < 0.2 ‰ for both δ ^13^C and δ ^15^N in 2016. Sample isotopic ratios were expressed in the conventional δ notation as parts per thousand (‰) according to the following equation:

1 $$\mathrm{\delta X }= [(\mathrm{Rsample}/\mathrm{Rstandard}) - 1] \times 1000$$

where X is ^13^C or ^15^N of the sample, and *R* is the corresponding ratio of ^13^C/^12^C or ^15^N/^14^N.

### Statistical analysis of stable isotope data

For the analysis of potential differences in benthic–pelagic coupling between sampling years, we included only station pairs that were geographically close to each other and located in similar bathymetric features (e.g., basin/ridge) (Fig. 5, Table 3), and contained either the same or closely related taxa (Table S2) in both years. To provide a general overview of the difference in isotopic niche structure between the two sampling years, bi-plots of δ^13^C versus δ^15^N were generated based on station-averaged values of each of the two carbon end-members (pPOM, sPOM) and each of the consumer groups (zooplankton and benthos). The isotopic niche widths of these four food web components (pPOM, sPOM, zooplankton, and benthos) were then calculated as Standard Ellipse Areas corrected for small size (SEA_c_)^97^. To compare the isotopic niches of food web components between years statistically, we used a Bayesian approach to calculate 100 000 posterior iterations of SEA^96,97^ that produced a range of probable SEAs (Bayesian SEA = SEA_B_) for each of the food web component from each year. This enabled robust statistical comparison of SEA_B_ between the sampling years by calculating the probability of difference between them^95,97^. Following^97^ and^98^, we considered a probability higher than 95% a meaningful difference. In addition, the overlap of SEAc of different food web components was calculated as the percentage of ellipse area shared by two components in order to test the hypothesis that benthic–pelagic coupling (expressed here as isotopic niche proximity) was tighter (= stronger overlap in SEAc) in 2005 than 2016. These analyses were conducted using the SIBER package (Stable Isotope Bayesian Ellipses in R;^95^) in R 4.0.3. statistical software^99^.

Isotopic distances of δ^15^N and δ^13^C between different food web components as a measure of pelagic-benthic coupling were calculated by subtracting the mean δ^15^N (δ^13^C) of one food web component from the mean δ^15^N (δ^13^C) of another food web component. This metric was used to test the hypothesis that distance between food web components was lower in 2005 than in 2016.

To test the hypothesis that δ^15^N was overall lower in benthos (reflecting fresher food reaching the seafloor through tighter pelagic-benthic coupling) and δ^13^C was higher (reflecting higher input of generally more ^13^C-enriched ice algae) in 2005 than in 2016, the means of δ^15^N and δ^13^C of each food web component were compared between the 2 years. The following tests were used for the comparison: a two-sample *t*-test (if distribution was normal and variances were equal), a Welch’s two sample *t*-test (if the distribution was normal, but the variances were not equal), and a Wilcoxon rank sum test (if the distribution was not normal). The Shapiro–Wilk test was applied to test for normality, followed by the Bartlett-test to verify the equality of variances. Values are presented as mean ± standard error (SE) in the text and tables. The analysis was conducted in R^99^.

## Supplementary Information

Supplementary Information 1.

Supplementary Information 2.

Supplementary Information 3.

## Data Availability

The datasets generated and analyzed for this study can be found in the https://mbon.ioos.us/#metadata/edc232ee-8582-4059-9c4c-7018b5af66a0/project.
