# Supplementary material for: Reduced efficiency of pelagic–benthic coupling in the Arctic deep sea during lower ice cover
Source: Sci Rep. 2023 Apr 25;13:6739. doi: 10.1038/s41598-023-33854-0 (PMC10130029; doi:10.1038/s41598-023-33854-0)
Supplement: Supplementary file 3 — Supplementary Information 3. [file 41598_2023_33854_MOESM3_ESM.docx]

**Reduced efficiency of pelagic-benthic coupling in the Arctic deep sea during lower ice cover**

Irina Zhulay, Katrin Iken, Paul E. Renaud, Ksenia Kosobokova, Bodil A. Bluhm

Table S3. Food web niche metrics for different food web components (pelagic particulate organic matter (pPOM), sediment POM (sPOM), zooplankton, and benthos) collected in the Chukchi Borderland in 2005 and 2016. Number of samples (*n*), average δ^13^C and δ^15^N ± standard error (‰), δ^13^C and δ^15^N range (‰), standard ellipse area corrected for small sample size (SEA_C_, ‰^2^) that includes 95% of the data, and modes of posterior probability distribution (SEA_B_).

|  | | n | Means | | Range | | SEAc | SEA_B_ |
| --- | --- | --- | --- | --- | --- | --- | --- | --- |
|  |  |  | δ^13^C | δ^15^N | δ^13^C | δ^15^N |  |  |
|  | **2005** | | | | | |  |  |
| pPOM | | 4 | - 25.6±0.7 | 3.4±0.5 | -24.0 to -27.7 | 1.4 to 5.1 ‰ | 3.0 | 2.7 |
| sPOM | | 5 | - 23.4±0.8 | 5.7±0.4 | -25.6 to -23.1 | 4.5 to 6.2 ‰ | 1.8 | 2.3 |
| Zooplankton | | 24 | - 22.2±0.3 | 13.1±0.4 | -27.8 to -19.7 | 8.1 and 17.2 ‰ | 10.0 | 9.4 |
| Benthos | | 20 | - 20.9±0.4 | 13.9 ± 0.6 | -24.8 to -16.6 | 10.4 to 19.5 ‰ | 16.8 | 15.6 |
|  | **2016** | | | | | |  |  |
| pPOM | | 7 | - 27.8±0.2 | 4.4±0.7 | -28.9 to -26.0 | 1.9 to 7.6 ‰ | 1.0 | 1.4 |
| sPOM | | 7 | - 21.5±0.1 | 7.6±0.4 | -22.4 to 20.4 | 4.7 to 10.2 ‰ | 1.9 | 0.8 |
| Zooplankton | | 28 | - 25.2±0.3 | 11.3±0.3 | -28.6 to -21.7 | 8.3 and 14.7 ‰ | 7.2 | 6.9 |
| Benthos | | 49 | - 20.2±0.3 | 16.5±0.3 | -24.6 to -16.2 | 12.4 to 20.5 ‰ | 9.1 | 8.9 |

Table S4. Overlap of SEA_C_ (%) calculated for SEAs, containing 95 % of data, of different food web components (pPOM, sPOM, zooplankton, and benthos) collected in the Chukchi Borderland in 2005 and 2016.

|  | pPOM_2005_ | sPOM_2005_ | Zooplankton_2005_ | Benthos_2005_ | pPOM_2016_ | sPOM_2016_ | Zooplankton_2016_ | Benthos_2016_ |
| --- | --- | --- | --- | --- | --- | --- | --- | --- |
| pPOM_2005_ | - |  |  |  |  |  |  |  |
| sPOM_2005_ | 4.8 | - |  |  |  |  |  |  |
| Zooplankton_2005_ | 0 | 0 | - |  |  |  |  |  |
| Benthos_2005_ | 0 | 0.1 | 57.9 | - |  |  |  |  |
| pPOM_2016_ | 3.6 | 0.9 | 0 | 0 | - |  |  |  |
| sPOM_2016_ | 0.4 | 1.8 | 2.6 | 3.7 | 0 | - |  |  |
| Zooplankton_2016_ | 0 | 0 | 21.0 | 23.3 | 0 | 0 | - |  |
| Benthos_2016_ | 0 | 0 | 27.6 | 50.7 | 0 | 0 | 5.5 | - |
